# Comparison of cervicovaginal fluid extracellular vesicles isolated from paired cervical brushes and vaginal swabs

**DOI:** 10.1002/jex2.153

**Published:** 2024-05-02

**Authors:** Emily Sarah Jane Paterson, Simon Scheck, Simon McDowell, Nick Bedford, Jane Eleanor Girling, Claire Elizabeth Henry

**Affiliations:** ^1^ Department of Surgery and Anaesthesia University of Otago Wellington, Aotearoa New Zealand; ^2^ Department of Obstetrics, Gynaecology and Women's Health University of Otago Wellington, Aotearoa New Zealand; ^3^ Department of Obstetrics and Gynaecology Wellington Hospital, Te Whatu Ora ‐ Capital, Coast and Hutt Valley Wellington, Aotearoa New Zealand; ^4^ Department of Anatomy University of Otago Dunedin, Aotearoa New Zealand

**Keywords:** biomarkers, cervical brush, cervicovaginal fluid, endometriosis, extracellular vesicles, vaginal swab

## Abstract

Endometriosis is a common gynaecological condition, with a long diagnostic delay. Surgery is required to confirm a diagnosis, highlighting the need for a non‐invasive biomarker. Extracellular vesicles (EVs) may have a role in endometriosis pathogenesis, yet there is limited EV biomarker literature available. This study aimed to investigate the feasibility of isolating cervico‐vaginal fluid EVs sampled using cervical brushes and vaginal swabs and to compare these methods. After providing informed consent, patients undergoing surgery for suspected endometriosis had cervical brush and vaginal swab samples collected under general anaesthetic. Isolated EVs were characterised through negative stain transmission electron microscopy (TEM), Western blotting (TSG101, CD63, Calnexin, ApoB, Albumin), tunable resistive pulse sensing (TRPS), microBCA assays and RT‐qPCR of miRNAs. PCR was performed on samples prior to EV isolation to assess bacteria present in samples. Cervical brush and vaginal swab EVs were intact vesicles with limited co‐isolated contaminants. Cervical brushes had higher concentrations of particles compared to match vaginal swabs, although both samples had low concentrations. Protein and miRNA yield were similar between matched samples. PCR demonstrated only a small amount DNA within samples was bacterial (>0.5%). Cervico‐vaginal fluids EVs were successfully isolated from cervical brushes and vaginal swabs, demonstrating a new method of sampling reproductive EVs. EV yield from both sample types was low. Similar protein and miRNA levels suggest either sampling method may be suitable for biomarker studies.

## 1 INTRODUCTION

Endometriosis is a disease defined by the presence of endometrial‐like tissue growing outside the uterus, causing symptoms such as chronic pelvic pain and reduced fertility. As such, endometriosis has a significant impact on quality of life (Facchin et al., 2015). With an estimated prevalence of one in nine women (while also affecting gender diverse people) (Rowlands et al., 2021), endometriosis also has a significant social and economic impact on society (Armour et al., 2019; Young et al., 2015).

Currently, gold standard diagnosis for endometriosis involves laparoscopic surgery with histopathology to confirm the presence of endometriotic lesions (ESHRE Endometriosis Guideline Development Group, 2022). Imaging such as ultrasound or MRI can help identify deep infiltrating endometriosis (DIE) or ovarian endometriosis (endometriomas), but superficial peritoneal endometriosis is generally unable to be visualised and so negative findings cannot rule out presence of the disease (ESHRE Endometriosis Guideline Development Group, 2022). Thus, confirming an endometriosis diagnosis requires surgical intervention, contributing to a diagnostic delay of over 6 years (Nnoaham et al., 2011). As such, there is a critical need for non‐invasive biomarkers of endometriosis to accelerate referrals from primary care, reducing healthcare costs and diagnostic delays.

Extracellular vesicles (EVs) are small lipid bilayer delimited particles that are released by cells into bodily fluids. EVs harbour molecular cargo from the cell of origin, and thus have garnered interest as promising biomarkers of disease. We and others (Liu et al., 2020), have previously reviewed current EV endometriosis research; most research available currently investigates the role of EVs in pathogenesis, and few papers have investigated EVs as biomarkers of endometriosis (Scheck et al., 2022).

Most current research using patient samples utilise EV sources that require invasive sampling such as peritoneal fluid (Chen et al., 2019; Lee et al., 2021; Nazri et al., 2020) or endometriosis tissue (Huang et al., 2022; Khalaj et al., 2019; Texidó et al., 2014). Indirect sources of EVs such as conditioned culture media (CCM) of ex‐vivo primary stromal or mesenchymal cells (Harp et al., 2016; Hsu et al., 2021; Qiu et al., 2020; Wu et al., 2018, 2020) are also often used, which are limited by the impact 2D culturing has on both the packaging of molecular cargo in to, and release of, EVs (Thippabhotla et al., 2019). Some have looked at serum and plasma EVs, which presumably contain very limited amounts of endometriosis or endometrium derived EVs, with only 0.2% of plasma EVs being tissue‐derived (Li et al., 2020). Thus, balancing the biological relevance of the EVs studied and the accessibility of sampling remains challenging.

We hypothesize that vaginal swabs and cervical brushes may strike the balance of accessibility and relevance. These samples may contain increased biologically relevant EVs for diseases of the female reproductive system such as endometriosis, compared to other biofluids like plasma and serum, while remaining a clinically standard sampling procedure. Thus far, EVs have been successfully isolated from cervical brushes (Luddi et al., 2019) and from a cervico‐vaginal swab in a single monkey (Muth et al., 2015). This feasibility study aimed to further investigate these sampling methods and compare the EV population isolated from matched cervical brushes and vaginal swabs, laying the foundation for further research into the potential of these samples for EV biomarkers of reproductive disease or dysfunction, including endometriosis.

## 2 METHODS

### 2.1 Ethical approval

This study was approved by the Central Health and Disability Ethics Committee (HDEC) (2022 EXP 12616), received endorsement from the Research Advisory Group—Māori (RAG‐M) at Te Whatu Ora—Capital and Coast (#937) and local approval from Te Whatu Ora—Capital and Coast research and audit committee. Consultation and feedback on patient information and consent forms was provided by the Endometriosis Research Community Advisory Group at the University of Otago Wellington.

### 2.2 Patient recruitment

People having surgery for suspected endometriosis at Te Whatu Ora—Capital and Coast (Wellington and Kenepuru Hospitals) were invited to participate in this study, and all patients provided informed written consent to participate. Eligible patients were aged between 16 and 45 years old. To limit EVs from less relevant tissue such as menstrual blood cells and EVs from possible malignancies, patients who were menstruating or had current known cervical abnormalities were excluded.

### 2.3 Sample collection and processing

Both vaginal swab and cervical brush samples were collected by the operating surgeon after the administration of anaesthesia, prior to surgical preparation. Sample collection media consisted of 1x phosphate buffered saline (PBS) (Fisher Scientific, #70‐011‐044) and contained 25 mM trehalose dihydrate (Sigma‐Aldrich, #T0167) and 25 mM Hepes (Thermo Scientific, #15630080).

Firstly, vaginal swabs were collected by inserting a FLOQ swab (Copan Diagnostics, #5E089N) into the vagina and turning for approximately 30 seconds, then placed into a tube with 1 mL collection media. Next, cervical brush samples were taken using a cervix brush (Rovers Medical Devices, #CMROV50) with use of a speculum, the brush head removed and placed in a tube containing 2 mL of collection media. Samples were visually assessed, and samples with any blood contamination were excluded. This included any samples with blood visible on the collection device, or if the sample fluid was orange or red, indicating blood had been introduced. All sample processing commenced within 1 h of collection.

To breakdown mucus and homogenise the sample, 20 mg/mL of N‐Acetyl‐L‐cysteine (Sigma‐Aldrich, #A9165) was added and the samples were incubated at 37°C for 15 min. The cervix brush or vaginal swab was removed from the tube, then the sample was centrifuged at 2000 × *g* at 4°C for 10 min to remove debris. The supernatant was transferred to a new tube and centrifugation was repeated. Samples were used immediately for EV isolation.

### 2.4 EV isolation

EVs were isolated from samples through size exclusion chromatography (SEC) using an automated fraction collector (Izon Science). Vaginal swab EVs were isolated using qEV1/70 nm columns (Izon Science, #IC1‐70) and four 0.7 mL fractions after the 4.7 mL buffer volume were collected, corresponding to fractions 6–9. Cervical brush EVs were isolated using qEV2/70 nm column (Izon Science, #SP4) and four 2 mL fractions were collected after the 12.1 mL buffer volume, corresponding to fractions 8–11.

EVs were then pooled in a Amicon 10 kDa 4 mL Centrifugal Filter Unit (Lab Supply NZ, UFC8010) and concentrated through centrifugation at 4000 × *g* at 4°C for 35 min. Concentrated EVs for RNA analysis, Western blotting and transmission electron microscopy (TEM) were kept on ice before subsequent preparation, while for Tunable Resistive Pulse Sensing (TRPS) and BCA assays concentrated EVs were stored at −80°C.

### 2.5 Tunable Resistive Pulse Sensing (TRPS)

The qNano Gold (Izon Science) was used to measure the EV concentration and determine the size profile of the EVs. The EV samples were measured on a Nanopore 200 (optimum range of 85–500 nm particles). Calibration particles with a 200 nm diameter (Izon Science, #CPC200) were diluted 1:200 to calibrate samples. Recording was stopped once a minimum particle of 500 was reached for calibration and sample measurement. If the minimum particle count was not reached throughout the 10 min of sample measurement the recording was stopped. Analysis of resulting data was conducted using the Izon Control Suite (version 3.3). Three matched cervical brush and vaginal swab were measured. The measured concentration was then adjusted for the concentration of samples post EV isolation, with the reported value as the concentration of particles in the original sample prior to EV isolation.

### 2.6 TEM

Negative staining was carried out at OMNI Electron Microscopy, University of Otago Dunedin. An aliquot of sample (10 µL) was placed onto a carbon coated 300 mesh copper grid. After 60 s the excess sample was removed by blotting and 10 µL of 1% phosphotungstic acid (PTA) in water was applied to the grid to contrast the sample. The PTA was blotted off immediately. The grids were viewed in a Philips CM100 BioTWIN transmission electron microscope (Philips/FEI Corporation), and images captured using a MegaView lll digital camera (Soft Imaging System GmbH).

### 2.7 Micro BCA assay

EV protein concentration was measured using the Pierce Micro BCA Protein Kit (Thermo Scientific, #23235) as per the manufacturer's instructions. Briefly, EVs were lysed in RIPA Buffer (Thermo Scientific, #89900) diluted 1:9 in distilled water containing protease inhibitor (Thermo Scientific, #A32953). Triplicates of 150 µL of lysed EVs, blank controls and albumin standards were plated. Working reagent was added to each well and the plate was incubated at 37°C for 2 h. Absorbance was measured at 562 nm on the Multiskan GO. Absorbance of the albumin standards were used to produce a standard curve which was used to determine the protein concentration of each sample.

### 2.8 Western blotting

Samples were tested for presence of EV markers CD63, TSG101 and for absence of contamination markers, ApoB, albumin and calnexin. For further concentration, EVs were added to a Amicon 10 kDa 0.5 mL Centrifugal Filter Unit (Lab Supply NZ, UFC5010) and centrifuged at 14,000 × *g* for 20 min, followed by a reverse spin at 1000 × *g* for 2 min to collect concentrate. Exosome Resuspension Buffer (from Total Exosome RNA & Protein Isolation Kit (Invitrogen, #4478545)) was added to make a final volume of 30 µL. Samples were thoroughly vortexed then incubated on ice for 30 min to lyse EVs.

For ApoB, Albumin, CD63 and Calnexin blots, EV lysate, cell lysate and plasma were added to LDS Sample Buffer (4x) (Invitrogen, #NP0007), for TSG101 EV lysate was added to Pierce™ Lane Marker Reducing Sample Buffer (Thermo Scientific, #39000), then heated at 75°C for five minutes. Positive controls were included for ApoB and Albumin (plasma diluted 1:100) and for Calnexin (endometrial cancer cell lysate) were included to demonstrate valid negative results.

EVs (35 µL) and Spectra Multicolour Broad Range Protein Ladder (10 µL, Thermo Scientific, #26634) were loaded on a 4–20% Tris‐Glycine gel (Invitrogen, #XP04200BOX). For ApoB and Albumin blots, 15 µL of plasma was also loaded. Gels were run in Tris Glycine SDS Running Buffer (Invitrogen, #LC2675) at 120 V for 90 min. Samples were transferred onto a 0.45 µm nitrocellulose membrane (Thermo Scientific, #88018) and blocked for 1 h (TSG101, CD63, Calnexin) or 3 h (ApoB, Albumin) at room temperature with 3% BSA (pH Scientific, #PH100) in 0.1% Tris‐buffered saline/Tween (TBST). Membranes were then incubated with the desired primary antibody (ApoB Thermo Fisher Scientific PA5‐86101, Albumin Santa Cruz Biotechnology sc‐271605) CD63 Santa Cruz Biotechnology sc‐5275, TSG101 Thermo Fisher Scientific PA5‐81094, Calnexin Thermo Fisher Scientific PA5‐86245) diluted in blocking solution (Albumin 1 µg/mL, CD63 1.33 µg/mL, ApoB, TSG101 and calnexin 5 µg/mL) overnight at 4°C. Membranes were washed with TBST, then were incubated with the appropriate secondary antibody (Mouse IgG Santa Cruz Biotechnology sc‐525409, Rabbit IgG Abcam ab205718) diluted in blocking solution (1:1000) for one hour at room temperature. Membranes were washed with TBST and then developed with the SuperSignal West Pico chemiluminescent substrate (Thermo Scientific, #34577) (Figure S1). Membranes were imaged using the iBright CL100 (Invitrogen). Band size was estimated visually using the protein ladder.

### 2.9 RNA extraction and RT‐qPCR

EV RNA was extracted using the Total Exosome RNA & Protein Isolation Kit (Invitrogen, #4478545) and RNA Clean & Concentrator‐5 (Zymo Research, #R1013). Briefly, using the Total Exosome Kit Exosome Resuspension Buffer was added to the concentrated EVs to make a final volume of 200 µL, followed by the addition of denaturing solution, then acid‐phenol chloroform, and subsequent centrifugation. The aqueous phase was transferred to a new tube and 1.25 volumes of 100% ethanol was added. The sample was then transferred to the Zymo‐Spin IC Column from the RNA Clean & Concentrator‐5 kit and centrifuged. RNA Prep Buffer, then two RNA Wash Buffer steps were carried out with centrifugation between each addition. RNA was eluted in 10 µL nuclease free water and stored at −80°C.

RNA was converted into cDNA using the TaqMan Advanced miRNA cDNA Synthesis Kit (Applied Biosystems, #A28007) as per manufacturer's instructions.

RT‐qPCR was carried out according to TaqMan Advanced miRNA Assays (Applied Biosystems, #A25576). Specific primers used and rationale for their selection are listed in Table S1. miR‐Amp cDNA was diluted 1:3 in nuclease free water before adding to each well. PCR cycling conditions were: 95°C for 20 s, followed by 40 cycles of 95°C for 3 s and 60°C for 30 s. Technical triplicates were averaged. PCR was performed on EV cDNA from three matched cervical brush and vaginal swab samples.

miR‐133a‐3p and miR‐363‐3p were used for normalisation of RT‐qPCR data. ΔC_t_ values were calculated by subtracting the mean of technical triplicate Ct values from the geomean of C_t_ values for miR‐133a and miR‐363. ΔΔC_t_ (ΔC_t_ VS–ΔC_t_ CB) values were calculated to find the difference in ΔC_t_ between matched cervical brush and vaginal swabs.

### 2.10 DNA extraction and PCR for bacterial genes

Cervical brush and vaginal swab samples were split in half, with one half incubated at 37°C for 15 min to replicate the incubation for EV isolation (N‐Acetyl‐L‐cysteine was not added due to interference with DNA extraction kit). DNA was then extracted using the Quick‐DNA Miniprep Plus kit (Zymo Research, #D4069) as per manufacturer's instructions. Briefly, 200 µL of biofluid buffer and 20 µL of 20 mg/mL proteinase K was added to 200 µL of sample and incubated for 10 min at 55°C. All centrifugation steps were at 12,000 × *g* for one minute at room temperature, unless otherwise stated. Samples were centrifuged to remove insoluble debris and the supernatant transferred to a new tube. Following the addition of one volume of genomic binding buffer, samples were transferred to spin columns and centrifuged. The following wash steps followed by centrifugation were conducted: 400 µL of DNA pre‐wash buffer, 700 µL gDNA wash buffer, 200 µL gDNA wash buffer. Columns were transferred to a new tube, with the addition of 50 µL of DNA elution buffer preheated to 60°C. Following a 5‐min incubation at room temperature, DNA was eluted through centrifugation. DNA concentration and purity was measured on the Nanodrop spectrophotometer (Thermo Fisher Scientific) with a 260/280 ratio of ∼1.8 considered pure. DNA was stored at −20°C until use. The qPCR assay was performed using the CFX384 Real‐Time PCR Detection System (BioRad). Each reaction contained 10 ng of DNA, 300 nM of universal primers targeting the 16S rRNA bacterial gene (Dorn‐In et al., 2015) (Forward: 5′ CAGCAGCCGCGGTAATAC 3′, reverse: 5′ ATCCTGTTTGMTMCCCVCRC 3′) (Integrated DNA Technologies) and 5 µL of SYBR Green FastMix (Quantabio, #95072‐012). Five different DNA quantities (0.005–1 ng) of a microbial DNA standard containing eight bacterial strains (Zymo Research, #D6305) was included. PCR cycling conditions were as follows: 95°C for 10 min, followed by 40 cycles of 95°C for 30 s, 60°C for 30 s, 72°C for 40 s, finishing with a final 60 s at 95°C followed by melt curve analysis. The C_t_ values were plotted against the log values of the five known microbial DNA quantities to create a standard curve to estimate total quantities of microbial DNA in the cervical brush and vaginal swab samples.

### 2.11 Statistics

Prism v9.2.0 (GraphPad) was used to calculate the mean and standard deviation for TRPS, RT‐qPCR, microBCA and bacterial DNA assay data. Two tailed paired *t*‐tests were performed on the ΔC_t_ values for each miRNA, DNA quantities of matched cervical brush and vaginal swab samples, and DNA quantities pre and post incubation, with significance set at *p* < 0.05.

## 3 RESULTS

There were 31 patients enrolled in the study. Seven patient samples were excluded due to presence of blood. Of these, two had blood in both cervical brush and vaginal swab samples and the remaining five had blood only in the cervical brush, however all were excluded due the need for matched swab and smear samples. The patient characteristics for the 24 patients included are listed in Table S2.

**FIGURE 1:**
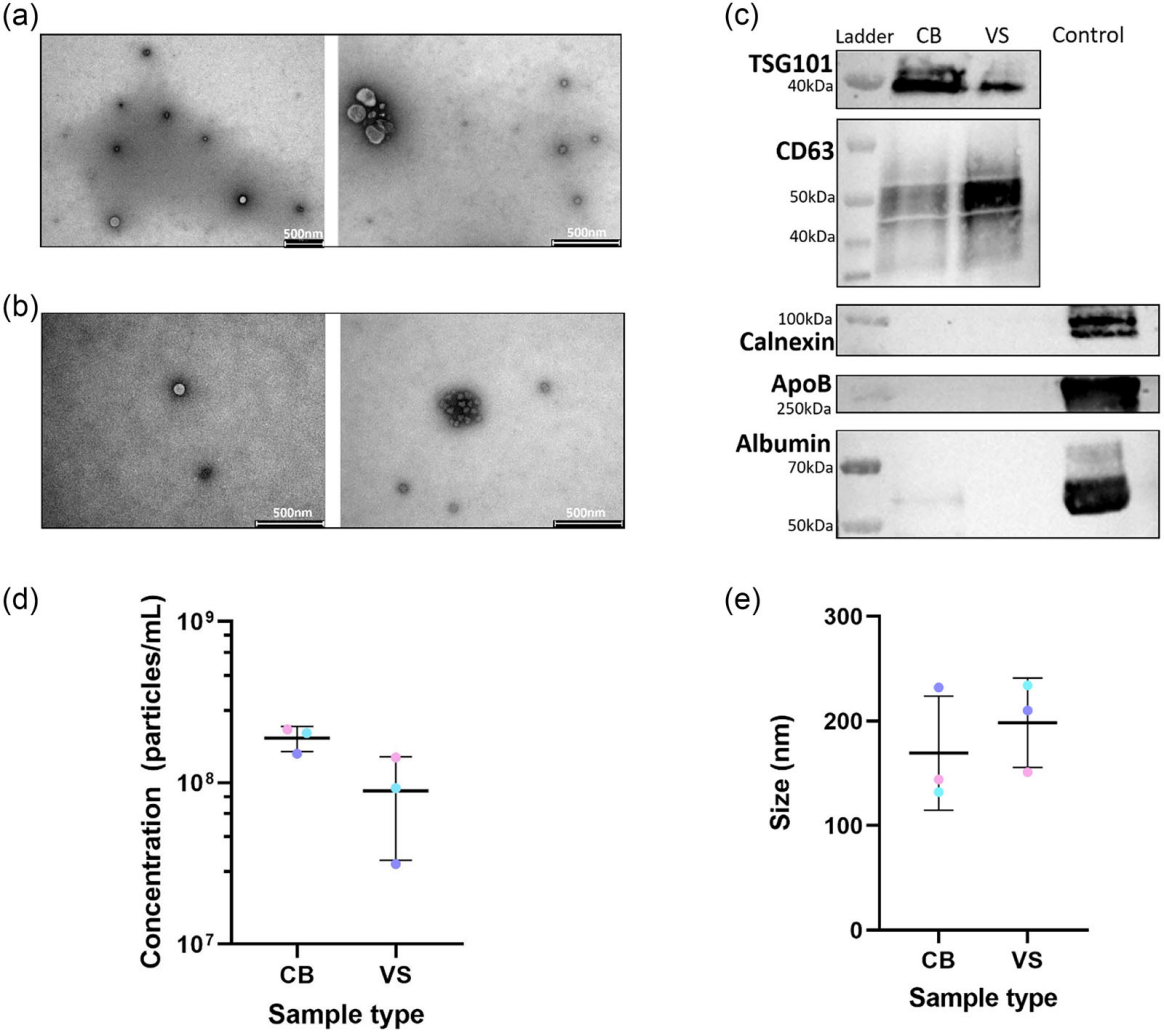
Characterisation of extracellular vesicles (EVs) isolated from cervical brush (CB) and vaginal swabs (VS). Negative stain transmission electron microscopy images of cervical brush (a) and vaginal swab (b) samples. (c) Western blotting of common EV markers and commonly co‐isolated contaminants, with size of protein ladder band annotated. Positive controls included plasma (diluted 1:100) for ApoB and Albumin blots and endometrial cancer cell lysate for Calnexin. Expected molecular weights: TSG101 – 44 kDa, CD63 – 25 kDa, Calnexin – 90 kDa, ApoB – 240 kDa, Albumin – 66 kDa. (d) Concentration and (e) mean size of particles measured by tunable resistive pulse sensing. *N* = 3, data presented as mean ± standard deviation, with individuals values plotted.

Isolated particles were round intact vesicular structures, as seen in representative negative stain TEM images (Figure 1a,b). Particles were sparse, with few particles per image, and there was a small amount of debris present.

Proteins TSG101, CD63 were detectable through Western blotting. There was no ApoB or calnexin detected in either sample type, there was a very small amount of albumin present in the cervical brush (Figure 1c). EV concentrations were higher in the cervical brush samples, with a mean of 1.89 × 10^8^ ± 3.32 × 10^7^ particles/mL compared to 8.90 × 10^7^ ± 5.60 × 10^7^ particles/mL in the vaginal swab samples (Figure 1d). Cervical brush samples showed less inter‐patient variability than vaginal swab samples. Mean particle size for vaginal swabs was higher than cervical brushes, at 200 ± 33 and 169 ± 45 nm, respectively (Figure 1e).

**FIGURE 2:**
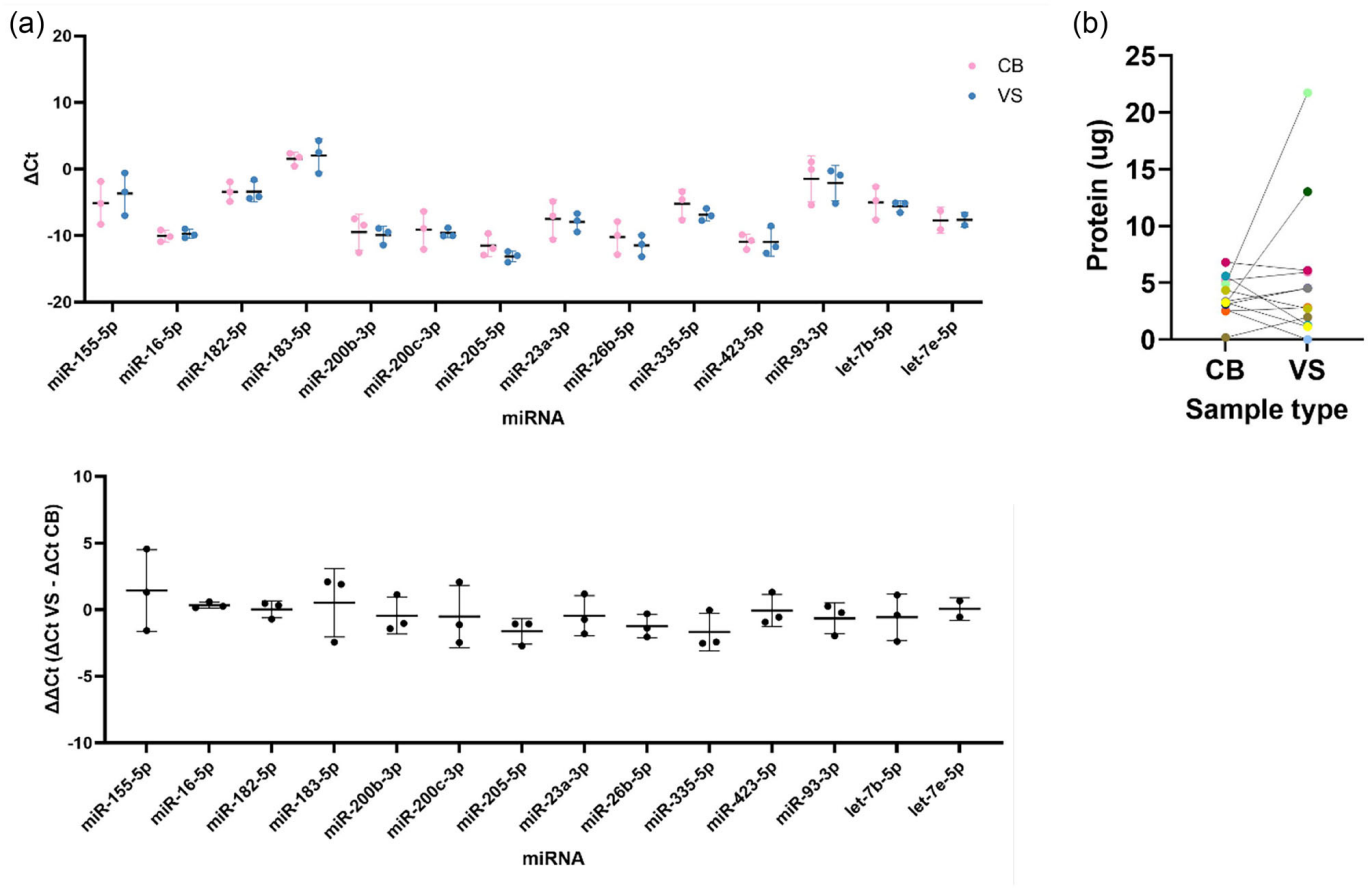
Assessment of RNA and protein content of cervical brush (CB) and vaginal swab (VS) extracellular vesicles (EVs). (a) Quantitative reverse transcription polymerase chain reaction (RT‐qPCR) of miRNA from matched cervical brush and vaginal swab EVs. ΔC_t_ values were calculated for each miRNA normalised to the geomean C_t_ values of miR‐133a‐3p and miR‐363‐3p. ΔΔC_t_ values were calculated by ΔC_t_ VS – ΔC_t_ CB to find the difference in normalised ΔC_t_ between VS and CBs. Data is presented as mean ± standard deviation (*n* = 3), with individual values plotted. (b) Total protein in matched cervical brush and vaginal swab samples after EV isolation as measured by microBCA assay (*n* = 12).

Of the 19 miRNAs analysed through RT‐qPCR, 16 were detectable in both vaginal swab and cervical brush samples. miR‐346 and miR‐504‐3p were not detectable in any samples, and miR‐25‐5p was detected only in one vaginal swab sample. The average of the biological triplicate ΔC_t_ values were similar between cervical brush and vaginal swab samples for all miRNAs measured, also reflected in low ΔΔC_t_ values which compare VS to CB ΔC_t_ values (Figure 2a), with no significant differences between cervical brushes and vaginal swabs (*p* > 0.05). Mean ± standard deviation C_t_ values for each miRNA are listed in Table S3.

The amount of protein in matched cervical brush and vaginal swab EV samples was similar, except for two patients who had increased protein within swab samples compared to cervical brushes (Figure 2b). The average protein content was slightly higher for vaginal swabs with 5.48 ± 5.90 µg compared to 3.76 ± 1.67 µg for cervical brushes.

**FIGURE 3:**
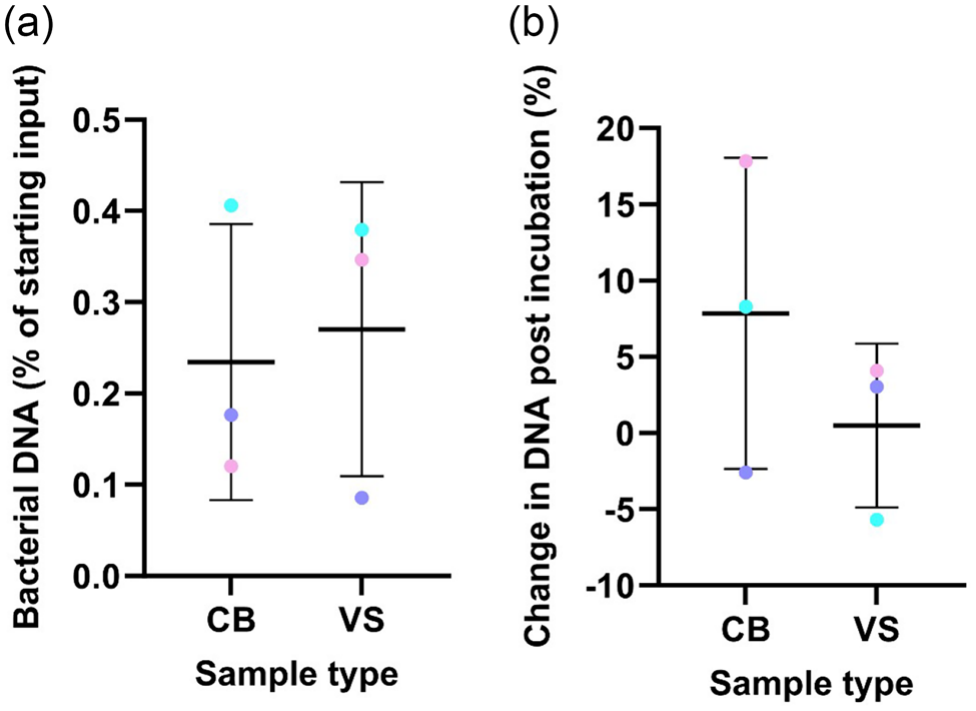
Assessment of bacterial DNA present within samples. (a) Bacterial DNA present within samples, presented as a percentage of input DNA (10 ng). (b) Effect of 15‐min incubation at 37°C on bacterial DNA, presented as percent difference between post‐incubation relative to pre‐incubation. Data are presented as mean ± standard deviation (*n* = 3), with individual values plotted.

The amount of bacterial DNA in the sample was minimal, with an average of 0.23 ± 0.15% of input DNA for cervical brushes and 0.27 ± 0.16% for vaginal swabs (Figure 3a). There was no significant difference between bacterial DNA quantities in matched cervical brushes and vaginal swabs (*p* > 0.05). There was no significant difference in bacterial DNA after the incubation period for cervical brushes or vaginal swabs (*p* > 0.05), and the percentage increase was 7.85 ± 10.21% for cervical brushes, and 0.48 ± 5.37% for vaginal swabs (Figure 3b).

## 4 DISCUSSION

In this study, we have established the feasibility of isolating EVs from vaginal swab and cervical brush samples. We found EVs can be successfully isolated from both samples, but the cervix brush had higher particle concentrations compared to the vaginal swab. We compared EV miRNA and protein from each sample type, finding that generally matched samples were comparable for both miRNA and protein levels.

The aim of this study was to compare our two chosen methods of sampling cervico‐vaginal fluid, vaginal swabs and cervical brushes. Both sample types are currently common practice in health care settings—vaginal swabs are commonly used for sexually transmitted infection (STI) self‐tests and cervical brushes are used for cervical cancer screening. Compared to surgical diagnosis for endometriosis, both methods would be attractive for diagnostics, both being cheap, fast and carrying minimal risk for the patient with no recovery time required. However, vaginal swabs provide further advantage with the ability to be self‐administered by the patient, while cervical brush samples require a speculum exam to be performed by a clinician. Blood derived EVs have also been investigated as biomarkers of endometriosis (Li et al., 2020; Shan et al., 2022; Zhang et al., 2020) however these EV populations are considerably heterogeneous due to the contribution of EVs from blood cells and tissues throughout the body. Research in other contexts has found only a small number of disease specific EVs are present within blood (Ricklefs et al., 2019). As such, bulk analysis of blood derived EVs may not be the most appropriate avenue for endometriosis EV biomarker research.

Based on the comparable miRNA and protein results between both swabs and smears, our data suggests that both sample types are comparable for EV biomarker studies. In terms of patient acceptability, in the context of cervical cancer screening human papillomavirus (HPV) testing, vaginal swabs have been found to be more acceptable compared to traditional cervical smears in a variety of populations around the world (Dzuba et al., 2002; Khoo et al., 2021), including Aotearoa New Zealand (Adcock et al., 2019; Brewer et al., 2021). As a result, cervical smears will be replaced with human papillomavirus (HPV) testing through vaginal swabs for routine screening in Aotearoa New Zealand, as well as in 47 other countries (Bruni et al., 2022). If vaginal swabs are to be considered for diagnosing endometriosis, the acceptability should be researched in people with endometriosis, as dyspareunia and other painful gynaecological symptoms are common in endometriosis (Ferrero et al., 2005) and as such may influence the acceptability.

We excluded people who were menstruating, or samples that had visible blood on the collection device or in the fluid component of the sample, to reduce the amount of blood cell EVs in the processed sample. For this pilot study, we attempted to limit the variables that could impact on our ability to compare matched samples to each other and samples between patients. This may reduce the clinical utility of this sampling method, if the requirement is for the sample to be clear; however, further research could determine the impact of the presence of blood on the EV population. The vaginal swabs were contaminated with blood less often than the cervical brushes, with only two of the excluded swab samples having visible blood contamination compared to all seven of the excluded cervical brush samples. This further supports the increased accessibility of the vaginal swab compared to cervical brush.

The amount of protein present within both cervical brush and vaginal swab samples was fairly low. This likely reflects the low EV yield found through TRPS analysis, and high sample purity as demonstrated with limited debris visible in TEM imaging and lack of contaminating proteins in Western blots. Attempts to maximise EV yield were made through the selection of the FLOQ Swabs which have superior sample elution compared to traditional rayon swabs (Daley et al., 2006), the choice of collection buffer to best preserve EVs (Görgens et al., 2022), isolating EVs from fresh samples and minimising the time between sample collection and processing. Differing EV isolation techniques could be trialled to investigate the impact on EV yield, as methodology impacts on the particle population isolated (Van Deun et al., 2014). SEC combined with ultrafiltration (UF), utilised in this study, is known to provide pure samples with good yields (Franco et al., 2023; Wei et al., 2020). However, the properties of the biofluid impact EV isolation efficiency (Momen‐Heravi et al., 2012), so further studies could investigate other isolation techniques to see if EV yield from cervico‐vaginal fluid could be further optimised. For example, ultracentrifugation (UC) does not require concentration of EVs through UF after SEC minimising EV loss through this process; however, the viscosity of the starting biofluid can impact the efficiency of EV isolation during UC (Momen‐Heravi et al., 2012) and needs to be considered. Alternatively, other techniques could be implemented such as taking multiple swabs/brushes to increase the cervico‐vaginal fluid sampled and subsequently the amount of EVs isolated.

Western blotting showed the EV population isolated from cervical brushes and vaginal swabs may be distinct, with differing levels of enrichment of EV markers TSG101 and CD63. The fluid collected by the cervical brush is likely comprised of primarily cervical mucus, while the vaginal swab may also contain secretions from the vaginal wall and Bartholin's glands. Furthermore, the microbiome of the vagina may also influence the EV population between sample types, with significant populations of bacteria within the vagina that likely release EVs into vaginal fluid, although in vivo research of vaginal bacterial EVs is very limited. The normal fluctuations of bacterial species throughout the menstrual cycle (Eschenbach et al., 2000), the impact of possible viral infections or dysbiosis (Wu et al., 2020), and inherent interpersonal microbiome differences between people (Ravel et al., 2011) all will likely impact the EV population isolated and are factors when considering biomarker studies. However, investigating the impact of these factors were outside the scope of this study, with the primary aim to determine the feasibility of isolating EVs from cervical brushes and vaginal swabs.

Only a small amount of the DNA within the samples collected was bacterial, and this was not affected significantly by the 15‐minute incubation at 37°C included in our methodology. This suggests that most of the sample collected was host derived, rather than bacterial derived. The microorganisms present within the female reproductive tract will presumably contribute to the EV population isolated, although with current technology available we are unable to quantify the proportion of EVs that are microbial derived. However, the bacterial EVs present may be an interesting avenue for future research, as there is emerging evidence of a potential link between the reproductive tract microbiome and endometriosis (Ata et al., 2019; Khan et al., 2010). The vaginal microbiome has also been investigated for diagnostic purposed, with Chao et al. finding the relative abundance of *Clostridium disporicum* and *Lactobacillus reuteri* bacterial species differed between people with endometriosis or adenomyosis, and people with chronic pelvic pain without endometriosis or adenomyosis, with a diagnostic sensitivity of 81% and specificity of 52% (Chao et al., 2021). This suggest that the contribution of EVs from bacteria present within the vagina may also provide a novel avenue to explore diagnostic EV biomarkers, rather than being a contaminating factor associated with vaginal swabs and cervical brush samples.

Of particular benefit, our method is significantly less invasive than many other methods previously used, increasing the accessibility of studying reproductive EVs. Commonly researched reproductive biofluids for EV research include follicular, uterine, and uterine tube fluids, and in EV endometriosis research peritoneal fluid. While of increased biological relevance, these samples often yield low volumes and require more invasive procedures resulting in challenges in acquiring these samples, particularly from patients (as opposed to animal models). Therefore, our described method holds potential for biomarker investigations of reproductive system pathologies such as endometriosis, cancers, and pregnancy related diseases such as pre‐eclampsia.

In this study, we used miRNAs miR‐133a‐3p and miR‐363‐3p to normalise RT‐qPCR data. These miRNAs were chosen as currently these miRNAs have not been identified as having differential expression in endometriosis, so were most appropriate for normalisation of data. However, it is important to note that these miRNAs may regulate endometrial cell processes important in endometriosis pathogenesis such as cell proliferation, adhesion and invasion (Li et al., 2018; Pan et al., 2017). For future research investigating EV miRNAs in cervical brushes or vaginal swabs, more in‐depth RNA analysis such as sequencing in a larger cohort of case and control samples could help to identify miRNAs that have stable abundance within cervico‐vaginal fluid EVs for normalisation strategies. Alternatively, a digital PCR approach could be taken to provide absolute quantification of miRNAs and removing the requirement of normalisation, which has been shown to improve reproducibility of EV RNA detection (Wang et al., 2019). Normalisation of EV miRNA PCR data is an ongoing challenge without standardised techniques, and as such we have reported both normalised and raw C_t_ values as supported by the MISEV guidelines (Welsh et al., 2024).

A strength of this study is the completion of basic EV characterisation, as described in the Minimal information for studies of extracellular vesicles 2018 (MISEV2018) guidelines (Théry et al., 2018). Our recent systematic review found that of endometriosis EV research, only around half of studies fully completed the basic characterisation required (Scheck et al., 2022).

A limitation of this study is that lack of menstrual cycle stage phenotyping for each patient. As the main aim of this study was to isolate EVs from matched cervical brush and vaginal swab samples, we did not collect samples exclusively from people in one phase of the menstrual cycle. In the future, the influence of menstrual cycle stage on the EVs isolated from cervical brush or vaginal swab samples should be investigated. Cervico‐vaginal fluid changes throughout the menstrual cycle in response to hormone fluctuations (Andersch‐Björkman et al., 2007), and thus EVs present within these fluids may also be affected by hormonal changes. Cell culture studies suggest that the concentration of endometrial EVs are unaffected by menstrual cycle, but molecular cargo is (Greening et al., 2016; Hart et al., 2023), and therefore controlling for menstrual cycle may be important for research into EV contents utilising this technique. Ultimately, an ideal biomarker of reproductive disease would be independent of menstrual phase, so this may depend on the goal of the investigations.

Another limitation of the study is the sole use of TRPS to estimate particle concentration. The NP200 pore used can analyse particles sized between 85 and 500 nm, so the ability to detect particles outside of this range is limited, possibly impacting on the accuracy of the concentration measurements. As EVs were isolated using 70 nm size exclusion columns (optimal recovery of particles 70–1000 nm), utilising the NP200 for particles concentration estimation was appropriate for this pilot study. Further research could employ multiple techniques of particle concentration estimation such as nanoparticle tracking analysis or nano flow cytometry to further interrogate the concentrations of particles from these sample types.

The bacterial DNA PCR assays included in this study were to provide an indication of how much bacterial contamination was present within these samples. This assay targeted the 16s RNA gene, and thus was non‐specific analysis. Other studies have thoroughly profiled the microbiome of the vagina through more specific approaches such as proteomics, and more in‐depth profiling was out of the scope of this study.

In this study, we have demonstrated the ability to isolate EVs from vaginal swabs and cervical brushes, providing a novel method of studying reproductive EVs. The EV yield was low, but there was measurable miRNA and protein present with comparable amounts for both sample types. Overall, we have illustrated the promise of our novel method of sampling EVs present in cervico‐vaginal fluids, which may hold potential for biomarkers of reproductive disease or dysfunction such as endometriosis.

## AUTHOR CONTRIBUTIONS

**Emily Sarah Jane Paterson**: Conceptualization (equal); data curation (lead); formal analysis (lead); investigation (lead); methodology (lead); project administration (lead); writing—original draft (lead); writing—review and editing (equal). **Simon Scheck**: Project administration (supporting); resources (equal); writing—review and editing (equal). **Simon McDowell**: Resources (equal); writing—review and editing (equal). **Nick Bedford**: Resources (equal); writing—review and editing (equal). **Jane Eleanor Girling**: Conceptualization (equal); supervision (equal); visualization (supporting); writing—review and editing (equal). **Claire Elizabeth Henry**: Conceptualization (equal); funding acquisition (lead); supervision (equal); visualization (supporting); writing—review and editing (equal).

## CONFLICT OF INTEREST STATEMENT

The authors declare no conflicts of interest.

## Supporting information

Supplementary Information
