# Supplementary material for: Comparison of cervicovaginal fluid extracellular vesicles isolated from paired cervical brushes and vaginal swabs
Source: J Extracell Biol. 2024 May 2;3(5):e153. doi: 10.1002/jex2.153 (PMC11080783; doi:10.1002/jex2.153)
Supplement: Supplementary file 1 — Supplementary Information [file JEX2-3-e153-s001.docx]

Appendices

Supplementary Table 1 Taqman Advanced miRNA Assay primers used for RT--qPCR with rationale for their selection

| Assay name | Assay ID | Rationale |
| --- | --- | --- |
| hsa--miR--133a--3p | 478511_mir | Used for normalisation of PCR data.  Caveat: expression drives endometrial epithelial cell proliferation (Pan et al., 2017). |
| hsa--miR--155--5p | 483064_mir | Increased in endometriotic lesions and eutopic endometrium compared to control eutopic endometrium (Brunty et al., 2021). |
| hsa--miR--16--5p | 477860_mir | Downregulated in serum of endometriosis patients compared to controls (Kumari et al., 2022). |
| hsa--miR--182--5p | 477935_mir | Upregulation inhibits proliferation, migration, invasion and inflammation in endometrial stromal cells (Wu et al., 2021). Decreased in endometriotic lesions compared to endometrium of cases, and in the endometrium of cases compared to controls (Jiang et al., 2020). |
| hsa--miR--183--5p | 477937_mir | Downregulated in endometriotic lesions compared to eutopic endometrium and in endometrium of cases compared to controls, regulates apoptosis of endometrial cells (Filigheddu et al. 2010, Shi et al., 2014). |
| hsa--miR--200b--3p | 477963_mir | Downregulated in endometriotic lesions compared to eutopic endometrium (Filigheddu et al. 2010, Hawkins et al., 2011) |
| hsa--miR--200c--3p | 478351_mir | Downregulated in endometriotic lesions compared to eutopic endometrium (Filigheddu et al. 2010, Hawkins et al., 2011, Shi et al., 2014) |
| hsa--miR--205--5p | 477967_mir | Downregulated in endometriotic lesions compared to eutopic endometrium from controls, and in serum between cases and controls, suppresses endometriosis progression *in vivo* (Zhou et al., 2019). |
| hsa--miR--23a--3p | 478532_mir | Downregulated in endometriotic lesions and eutopic endometrium of cases compared to endometrium of controls (Shen et al., 2013). |
| hsa--miR--25--5p | 478786_mir | Downregulated in endometriotic lesions compared to eutopic endometrium (Filigheddu et al. 2010, Shi et al., 2014). |
| hsa--miR--26b--5p | 478418_mir | Downregulated in serum EVs of endometriosis cases compared to controls (Wu et al., 2022). |
| hsa--miR--335--5p | 478324_mir | Downregulated in serum of cases compared to controls (Wang et al., 2013). |
| hsa--miR--346 | 478046_mir | Regulates EG-VEGF (Su et al., 2017), important in angiogenesis of endometriotic lesions (Lee et al., 2010). |
| hsa--miR--363--3p | 478060_mir | Used for normalisation of PCR data.  Caveat: associated with migration, cell adhesion, invasion, proliferation and apoptosis in endometrial stromal cells *in vitro* (Li et al., 2018). |
| hsa--miR--423--5p | 478090_mir | Upregulated in serum compared to controls (Hsu et al., 2014), and variant rs6505162A>C significantly associated with endometriosis (Jaafar et al., 2022). |
| hsa--miR--504--3p | 478956_mir | Downregulated in endometriomas compared to control endometrium (Hawkins et al., 2011). |
| hsa--miR--93--3p | 478209_mir | Downregulated in endometriotic lesions compared to eutopic endometrium (Shi et al., 2014), upregulated in eutopic endometrium of cases compared to controls (Antonio et al., 2023). |
| hsa--let--7b--5p | 478576_mir | Downregulated in serum of cases compared to controls (Moustafa et al., 2020). |
| hsa--let--7e--5p | 478579_mir | Downregulated in serum of cases compared to controls in the proliferative phase (Cho et al., 2015). |

Supplementary Table 2 Demographic and clinical characteristics of patients undergoing surgery for suspected endometriosis

| Characteristic | N (%) |
| --- | --- |
| Age |  |
| 30.6 ± 7.6 | N/A |
| Ethnicity |  |
| New Zealand European | 11 (45.8%) |
| Māori | 6 (25.0%) |
| Asian | 3 (12.5%) |
| Other European | 2 (8.3%) |
| African | 1 (4.2%) |
| Indian | 1 (4.2%) |
| Endometriosis diagnosis |  |
| Yes | 16 (67%) |
| No | 8 (33%) |
| Symptoms experienced |  |
| Painful periods | 24 (100%) |
| Pelvic pain unrelated to periods | 20 (85.7%) |
| Painful bowel motions | 20 (85.7%) |
| Other bowel problems | 22 (90.5%) |
| Painful intercourse | 19 (85.0%) |
| Difficulty falling pregnant | 8 (35.0%) |
| Abnormal menstrual bleeding | 18 (81.0%) |
| Other period related symptoms | 14 (61.9%) |
| Hormonal medications |  |
| Yes | 9 (37.5%) |
| No | 15 (62.5%) |
| Periods in the last three months |  |
| Yes | 17 (70.8%) |
| No | 7 (29.2%) |
| N = 24  Age is presented as mean ± standard deviation | |

Supplementary Table 3 Mean C_t_ values of miRNAs measured by RT--qPCR

|  | Mean C_t_ value of biological triplicates | |
| --- | --- | --- |
| miRNA | CB | VS |
| miR--133a--3p | 33.83 ± 2.83 | 33.01 ± 0.53 |
| miR--155--5p | 26.56 ± 2.41 | 28.43 ± 2.75 |
| miR--16--5p | 21.57 ± 4.75 | 22.35 ± 0.17 |
| miR--182--5p | 28.25 ± 3.48 | 28.72 ± 1.93 |
| miR--183--5p | 33.18 ± 3.75 | 34.14 ± 3.09 |
| miR--200b--3p | 22.17 ± 2.05 | 22.17 ± 1.33 |
| miR--200c--3p | 22.54 ± 2.39 | 22.47 ± 1.17 |
| miR--205--5p | 20.15 ± 4.56 | 18.96 ± 1.40 |
| miR--23a--3p | 24.15 ± 2.19 | 24.14 ± 1.15 |
| miR--25--5p | ND | ND |
| miR--26b--5p | 21.42 ± 2.56 | 20.62 ± 1.73 |
| miR--335--5p | 26.43 ± 2.60 | 25.21 ± 1.19 |
| miR--346 | ND | ND |
| miR--363--3p | 26.04 ± 0.79 | 29.23 ± 2.73 |
| miR--423--5p | 20.75 ± 5.65 | 21.14 ± 2.35 |
| miR--504--3p | ND | ND |
| miR--93--3p | 30.19 ± 1.32 | 29.99 ± 2.39 |
| let--7b--5p | 26.64 ± 2.55 | 26.52 ± 0.82 |
| let--7e--5p | 25.42 ± 3.64 | 24.27 ± 0.40 |
| CB – cervical brush, VS – vaginal swab, ND – not detected | | |

**References**

Antonio, L. G. L., Meola, J., Rosa‐e‐Silva, A. C. J. D. S., Nogueira, A. A., Candido dos Reis, F. J., Poli‐Neto, O. B., & Rosa‐e‐Silva, J. C. (2023). Altered differential expression of genes and microRNAs related to adhesion and apoptosis pathways in patients with different phenotypes of endometriosis. International Journal of Molecular Sciences, 24(5), 4434.

Brunty, S., Ray Wright, K., Mitchell, B., & Santanam, N. (2021). Peritoneal modulators of EZH2‐miR‐155 cross‐talk in endometriosis. International Journal of Molecular Sciences, 22(7), 3492.

Cho, S., Mutlu, L., Grechukhina, O., & Taylor, H. S. (2015). Circulating microRNAs as potential biomarkers for endometriosis. Fertility and Sterility, 103(5), 1252–1260.

Filigheddu, N., Gregnanin, I., Porporato, E., Surico, D., Perego, B., Galli, L., Patrignani, C., Graziani, A., & Surico, N. (2010). Differential expression of microRNAs between eutopic and ectopic endometrium in ovarian endometriosis. Journal of Biomedicine and Biotechnology, 2010, 369549.

Hawkins, S. M., Creighton, C. J., Han, D. Y., Zariff, A., Anderson, M. L., Gunaratne, H., & Matzuk, M. M. (2011). Functional microRNA involved in endometriosis. Molecular Endocrinology, 25(5), 821–832.

Hsu, C. Y., Hsieh, T. H., Lin, H. Y., Lu, C. Y., Lo, H. W., Tsai, C. C., & Tsai, E. M. (2021). Characterization and proteomic analysis of endometrial stromal cell–derived small extracellular vesicles. The Journal of Clinical Endocrinology & Metabolism, 106(5), 1516–1529.

Hsu, C. Y., Hsieh, T. H., Tsai, C. F., Tsai, H. P., Chen, H. S., Chang, Y., Chuang, H. Y., Lee, J. N., Hsu, Y. L., & Tsai, E. M. (2014). miRNA‐199a‐5p regulates VEGFA in endometrial mesenchymal stem cells and contributes to the pathogenesis of endometriosis. The Journal of Pathology, 232(3), 330–343.

Jaafar, S. O., Jaffar, J. O., Ibrahim, S. A., & Jarjees, K. K. (2022). MicroRNA Variants miR‐27a rs895819 and miR‐423 rs6505162, but not miR‐124‐1 rs531564, are linked to endometriosis and its severity. British Journal of Biomedical Science, 79, 10207.

Jiang, L., Zhang, M., Wang, S., Xiao, Y., Wu, J., Zhou, Y., & Fang, X. (2020). LINC01018 and SMIM25 sponged miR‐182‐5p in endometriosis revealed by the ceRNA network construction. International Journal of Immunopathology and Pharmacology, 34, 2058738420976309.

Kumari, P., Sharma, I., Saha, S. C., Srinivasan, R., & Bhardwaj, P. (2022). Role of serum microRNAs as biomarkers for endometriosis, endometrioid carcinoma of ovary & endometrioid endometrial cancer. Indian Journal of Medical Research, 156(3), 516–523.

Lee, K. F., Lee, Y. L., Chan, R. W., Cheong, A. W., Ng, E. H., Ho, C., & Yeung, W. S. (2010). Up‐regulation of endocrine gland‐derived vascular endothelial growth factor but not vascular endothelial growth factor in human ectopic endometriotic tissue. Fertility and Sterility, 93(4), 1052–1060.

Li, W., Fan, X., Zhang, M., Huang, L., Lv, S., Wang, L., Wu, Y., Dai, C., Xu, J., Xu, & Fu, Z. (2018). Systematic analysis of hsa‐miR‐363 gene overexpression pattern in endometrial stromal cells. International Journal of Molecular Medicine, 42(5), 2793–2800.

Moustafa, S., Burn, M., Mamillapalli, R., Nematian, S., Flores, V., & Taylor, H. S. (2020). Accurate diagnosis of endometriosis using serum microRNAs. American Journal of Obstetrics and Gynecology, 223(4), 557 e1.

Pan, J. L., Yuan, D. Z., Zhao, Y. B., Nie, L., Lei, Y., Liu, M., Long, Y., Zhang, J. H., Blok, L. J., Burger, C. W., & Yue, L. M. (2017). Progesterone‐induced miR‐133a inhibits the proliferation of endometrial epithelial cells. Acta Physiologica, 219(3), 685–694.

Shen, L., Yang, S., Huang, W., Xu, W., Wang, Q., Song, Y., & Liu, Y. (2013). MicroRNA23a and microRNA23b deregulation derepresses SF‐1 and upregulates estrogen signaling in ovarian endometriosis. The Journal of Clinical Endocrinology & Metabolism, 98(4), 1575–1582.

Shi, X. Y., Gu, L. I. N., Chen, J. I. E., Guo, X. R., & Shi, Y. L. (2014). Downregulation of miR‐183 inhibits apoptosis and enhances the invasive potential of endometrial stromal cells in endometriosis. International Journal of Molecular Medicine, 33(1), 59–67.

Su, M. T., Tsai, Y., Tsai, H. L., Chen, Y. C., & Kuo, L. (2017). miR‐346 and miR‐582‐3p‐regulated EG‐VEGF expression and trophoblast invasion via matrix metalloproteinases 2 and 9. Biofactors, 43(2), 210–219.

Wang, W. T., Zhao, Y. N., Han, B. W., Hong, S. J., & Chen, Y. Q. (2013). Circulating microRNAs identified in a genome‐wide serum microRNA expression analysis as noninvasive biomarkers for endometriosis. The Journal of Clinical Endocrinology & Metabolism, 98(1), 281–289.

Wu, M., & Zhang, Y. (2021). MiR‐182 inhibits proliferation, migration, invasion and inflammation of endometrial stromal cells through deactivation of NF‐κB signaling pathway in endometriosis. Molecular and Cellular Biochemistry, 476, 1575–1588.

Wu, Y., Yuan, W., Ding, H., & Wu, X. (2022). Serum exosomal miRNA from endometriosis patients correlates with disease severity. Archives of Gynecology and Obstetrics, 305(1), 117–127.

Zhou, C. F., Liu, M. J., Wang, W., Wu, S., Huang, Y. X., Chen, G. B., Liu, L. M., Peng, D. X., Wang, X. F., Cai, X. Z., & Li, X. X. (2019). miR‐205‐5p inhibits human endometriosis progression by targeting ANGPT2 in endometrial stromal cells. Stem Cell Research & Therapy, 10(1), 287.
